# Changes in Brain Glutamate on Switching to Clozapine in Treatment-Resistant Schizophrenia

**DOI:** 10.1093/schbul/sbaa156

**Published:** 2021-01-05

**Authors:** Grant McQueen, Kyra-Verena Sendt, Amy Gillespie, Alessia Avila, John Lally, Kalliopi Vallianatou, Nynn Chang, Diogo Ferreira, Faith Borgan, Oliver D Howes, Gareth J Barker, David J Lythgoe, James M Stone, Philip McGuire, James H MacCabe, Alice Egerton

**Affiliations:** 1 Department of Psychosis Studies, Institute of Psychiatry, Psychology & Neuroscience, King’s College London, De Crespigny Park, London, UK; 2 Department of Psychiatry, Royal College of Surgeons in Ireland, Dublin, Ireland; 3 Hospital de Santa Maria, Centro Hospitalar Universitário de Lisboa Norte, Lisbon, Portugal; 4 Department of Neuroimaging, Centre for Neuroimaging Sciences, Institute of Psychiatry, Psychology & Neuroimaging, King’s College London, De Crespigny Park, London, UK; 5 South London and Maudsley NHS Trust, London, UK

**Keywords:** ^1^H-MRS, magnetic resonance spectroscopy, antipsychotic, anterior cingulate cortex, caudate, psychosis

## Abstract

It has been suggested that the antipsychotic clozapine may modulate brain glutamate, and that this effect could contribute to its efficacy in treatment-resistant schizophrenia (TRS). The aim of this study was to examine the effects of clozapine on brain glutamate in TRS longitudinally. This study examined individuals with TRS before and 12 weeks after switching from a non-clozapine antipsychotic to treatment with clozapine as part of their normal clinical care. Proton magnetic resonance spectroscopy (^1^H-MRS) measured concentrations, corrected for voxel tissue content, of glutamate (Glu_corr_), and glutamate plus glutamine (Glx_corr_) in the anterior cingulate cortex (ACC) and right caudate nucleus. Symptoms were monitored using the Positive and Negative Syndrome Scale (PANSS). Of 37 recruited patients (27 men, 39.30 years old, 84% clozapine naïve), 25 completed ^1^H-MRS at both timepoints. 12 weeks of clozapine was associated with a longitudinal reduction in Glu_corr_ in the caudate (*n* = 23, *F* = 7.61 *P* = .01) but not in the ACC (*n* = 24, *F* = 0.02, *P* = .59). Percentage reduction in caudate Glu_corr_ was positively correlated with percentage improvement in symptoms (total PANSS score, *n* = 23, *r* = .42, *P* = .04). These findings indicate that reductions in glutamate in the caudate nucleus may contribute to symptomatic improvement during the first months of clozapine treatment.

## Introduction

Clozapine is the only antipsychotic recommended for patients with treatment-resistant schizophrenia (TRS).^1^ However, approximately half of patients with TRS do not respond to clozapine.^2,3^ The neurobiological mechanisms that mediate clozapine response are unclear, and it is currently not possible to predict in advance whether clozapine will be effective in improving symptoms.

Cross-sectional proton magnetic resonance spectroscopy (1H-MRS) studies indicate that patients with TRS taking non-clozapine antipsychotics have higher levels of glutamate in the anterior cingulate cortex (ACC) than patients with schizophrenia who have shown a good antipsychotic response^4^ and healthy volunteers.^5,6^ Similarly, elevated ACC glutamate levels have been linked to a poor response to conventional antipsychotic treatment early in illness.^7,8^ In contrast, studies examining participants with TRS taking clozapine at the time of scanning have reported no difference in ACC glutamate or Glx (the combined signal from glutamate and glutamine) in comparison to an antipsychotic-responsive group.^9,10^ One explanation for this difference could be that clozapine treatment reduces ACC glutamate levels. Interestingly, binding to the NMDA receptor intrachannel PCP/MK-801 site is reduced across the brain in clozapine-treated, but not typical-antipsychotic-treated patients with schizophrenia relative to healthy volunteers, which may also indicate that clozapine modulates glutamatergic systems.^11^ It is also possible that a reduction in ACC glutamate may relate to the degree of the clozapine response. In comparison to healthy volunteers, ACC Glx levels were elevated in clozapine-nonresponsive TRS (termed ultra-resistant schizophrenia, URS), but not in patients with TRS who had responded to clozapine.^10^

In addition to the ACC, schizophrenia is associated with glutamatergic elevation in the striatum.^12^ In first-episode psychosis, effective treatment with risperidone reduces glutamate in the right caudate (associative striatum) and this reduction correlates with improvement in symptoms.^13^ It is unknown whether effective clozapine treatment produces similar reductions in striatal glutamate. One cross-sectional study including patients with TRS taking clozapine found higher Glx in the putamen compared to both an URS and an antipsychotic-responsive group,^9^ although no group difference was found in a similar study examining the caudate.^10^ Overall, while effective treatment with non-clozapine antipsychotics may reduce glutamate or Glx in the ACC^14–16^ and striatum^13^ (and see^17,18^), it is possible that glutamate metabolites remain elevated in patients who are treatment-resistant. The effects of clozapine on glutamate in either the ACC or striatum have not been yet investigated longitudinally in humans.

While ^1^H-MRS measures the total amount of intracellular glutamate in the voxel and not that associated with neurotransmission specifically, effects of clozapine on glutamatergic neurotransmission have been described in preclinical research. In the rodent prefrontal cortex, clozapine attenuates the increases in extracellular glutamate efflux^19–21^ and disruptions in neuronal firing^22^ that are stimulated by blocking N-methyl-D-aspartate (NMDA) receptors. In rodents, clozapine administration also decreases glutamate concentrations in ex vivo frontal cortex tissue samples,^23,24^ and may decrease the in vivo ^1^H-MRS glutamate signal in the rat striatum.^25^

Whilst the effects of clozapine on brain glutamate in schizophrenia have not been investigated before, clinical studies have examined the effect of clozapine on plasma or serum glutamate levels. These studies have reported increases,^26^ decreases^27^ and no effect^28,29^ of clozapine administration overall. However, a good response has recently been associated with higher serum glutamate levels relative to poor clozapine response.^30^ Nonetheless, the relationship between peripheral and central glutamate levels is unclear and the role of glutamate in the clozapine response can be more directly evaluated by using ^1^H-MRS to measure brain glutamate levels in patients undergoing clozapine treatment.

The aim of this study was to measure glutamate levels in the ACC and striatum in patients with TRS before and 12 weeks after switching from their current antipsychotic to treatment with clozapine. Our main hypothesis was that glutamate would be reduced in these 2 areas over the 12-week observation period. In secondary analyses, we hypothesized that longitudinal reductions in glutamate concentration would be correlated with the degree of symptomatic improvement.

## Methods

### Participants and Clinical Measures

This study was approved by London South East NHS ethics committee (Ref:13/LO/1857). Participants were recruited from inpatient and outpatient services within the South London and Maudsley, and the Oxleas NHS Foundation Trusts.

The study included participants meeting ICD-10 criteria for schizophrenia (F20) or schizoaffective disorder (F25), as diagnosed by their treating psychiatrist, who were due to switch from their current antipsychotic to clozapine as part of their normal clinical care. Inclusion required that participants were clozapine naïve or had not taken clozapine for at least 3 months prior to the study. The presence of TRS was inferred from medical records and from their treating psychiatrist. Criteria included at least 2 previous trials of a non-clozapine antipsychotic, each within the recommended dose range for at least 6 weeks, a diagnosis of TRS provided by their psychiatrist and referral for clozapine initiation. Participants with mental capacity to consent provided written informed consent to study procedures. The study was also open to participants lacking capacity to consent if a consultee advised assent on their behalf. The consultee was defined as a person nonprofessionally involved in caring for the patient or concerned with their welfare (normally their next-of-kin family member). They were advised on the role of the consultee, provided with a consultee information sheet and invited to ask any questions about the study before advising on the patient’s behalf. If there was any indication from the patient, consultee, clinical team, or anyone else involved in the patient’s care that the patient would not wish to participate, they were not enrolled in the study. During study participation, study researchers maintained contact with the consultee and clinical team. If the patient expressed any objections to the study or wishes to withdraw, they were withdrawn from the study immediately. General exclusion criteria included drug dependency, as defined in DSM-IV, or pregnancy. Participants with contraindications to magnetic resonance imaging (MRI) at 3 Tesla were excluded from MRI but were able to participate in the larger study, including clinical assessment and blood sampling.

Clinical assessment and MRI/MRS were performed at baseline (−14 to 0 days before clozapine titration) and repeated 12 weeks after clozapine initiation. This period was selected as a 12-week treatment period has been associated with clozapine response in ~50% of patients.^31,32^ Clozapine plasma levels were measured in blood samples taken at 6 and 12 weeks to evaluate whether clozapine levels were above the indicated therapeutic threshold of 350 ng/ml,^32^ blood samples were also collected at 8 weeks if below threshold at 6 weeks.

Demographic and clinical history was collected through self-report and review of medical records. Clinical assessment instruments included the Positive and Negative Syndrome Scale (PANSS),^33^ Clinical Global Impression – Schizophrenia scale (CGI-S)^34^ Global Assessment of Functioning scale (GAF)^35^ and Personal and Social Performance scale (PSP).^36^ We assessed the percentage change in PANSS total score from baseline to 12 weeks using the formula: (12-week score – baseline score) / baseline score × 100, after subtracting minimum possible scores.^37^

### Magnetic Resonance Imaging

MR data were acquired on a 3 Tesla MR750 scanner (General Electric) (supplementary methods). ^1^H-MRS spectrum were acquired in 8 cm^3^ (2 × 2 × 2 cm^3^) voxels prescribed in the bilateral ACC^7,8^ and in the right caudate nucleus^13,38^ (supplementary figure S1). Spectrum were acquired using a conventional PRESS (Point RESolved Spectroscopy) acquisition with 96 averages, TR = 3000 ms and with a TE = 30 ms in the ACC and a TE = 35 ms in the caudate.^13^

### Data Processing

Spectrum were analyzed with LCModel version 6.3-0I (supplementary methods). Spectral quality was evaluated by visual inspection, and individual metabolite estimates were excluded if the Cramér Rao Lower Bound estimates of the standard deviations (%SD) were greater than >20%.

Metabolite values were corrected for voxel tissue content using the formula:

$$\begin{array}{l} Mcorr\;=\;M*(WM\;+\;1.21*GM\;+\;1.55*CSF)\;/\;\left(wm\;+\;gm\right), \end{array}$$

where M is the uncorrected metabolite, and WM, GM and CSF indicate the fraction of white and gray matter and cerebrospinal fluid content in the voxel. The formula assumes a CSF water concentration of 55 556 mol/m^3^.^39,40^ Voxel GM ratio was calculated as GM/(GM+WM).

### Statistical Analysis

Analysis was performed in SPSS version 25, IBM. Data were inspected for normality of distribution and outlying values. Initial analysis identified any relationships between glutamatergic metabolite levels at baseline and the clinical and demographic characteristics of the sample using *t*-tests for categorical variables and Pearson’s correlations for continuous variables (threshold *P* = .05). Where significant, the potential influences of these variables were subsequently investigated during hypothesis testing. For the main analysis, repeated measures analysis of variance examined the effects of 12 weeks of clozapine treatment on Glu_corr_ and Glx_corr_ levels in the ACC and caudate. In the main analysis, the threshold for statistical significance was corrected for the 2 voxels examined (*P* = 0.05/2 = .025). In exploratory analysis, Pearson’s correlations investigated relationships between glutamate metabolite levels and PANSS Total scores, and between the percentage change in glutamate metabolites and PANSS scores over 12 weeks.

## Results

### Demographic and Clinical Characteristics of the Sample

Seventy-seven participants with TRS consented to the larger study, of whom 37 completed at least one MR session (table 1). Common reasons for nonparticipation in MR included presence of MRI contraindication, being above the scanner weight limit, or being too unwell to leave the ward or tolerate the scanning session. Within patients in whom baseline PANSS scores were available, there was no significant difference in symptom severity between those who did or did not participate in MRI (mean ± SD PANSS total score: MRI: *n* = 37; 79.19 ± 15.02; No MRI: *n* = 24; 74.21 ± 19.44; *t*(59) = −0.46; *P* = .65). Twenty-five participants had complete data, including ^1^H-MRS and PANSS scores at both baseline and 12 weeks. A further 10 participants completed ^1^H-MRS at baseline (total baseline ^1^H-MRS = 35), of whom 7 subsequently left the study, and 3 completed 12-week PANSS but not 12-week ^1^H-MRS. An additional 2 participants declined baseline ^1^H-MRS but completed all PANSS ratings and 12-week MRS (total 12-week ^1^H-MRS = 27). This resulted in PANSS ratings in a total of 37 patients at baseline, and 30 patients at 12 weeks. Reasons for drop-out over the observation period (total *n* = 7), included not commencing clozapine (*n* = 2), discontinuing clozapine due to poor tolerability or other medical concern (*n* = 4) or moving outside the participating NHS Trusts (*n* = 1). Concomitant use of GABA-acting (eg, benzodiazepines) and/or serotonergic (eg, selective serotonin reuptake inhibitor antidepressants) was relatively common (32% and 51% of the sample, respectively, table 1), as were psychiatric comorbidities and previous substance use (table 1).

**Table 1.** Demographic and Clinical Characteristics of the Total Sample

Age	39.30 ± 13.56
Sex (M / F)	27 / 10
Age of onset	25.51 ± 8.74
Duration of illness	15.08 ± 9.19
Previous antipsychotic trials (median; range; min; max)	3; 8; 2; 10
Previous clozapine trial (Y / N)	6 / 31
Number of hospitalizations (median; range; min; max)	3; 12; 0; 12
Capacity to consent (Y/N)	32 / 5
Diagnosis (F20 / F25)	30 / 7
Comorbid psychiatric diagnosis	
Affective disorder	6
Anxiety disorder	1
Epilepsy	1
Personality disorder	1
Post-traumatic stress disorder	1
None	27
Antipsychotic before switching to clozapine (N):	
Olanzapine	10
Aripiprazole	5
Amisulpride	4
Risperidone	5
Quetiapine	4
Flupentixol	2
Asenapine	1
Haloperidol	1
Zuclopenthixol	1
Antipsychotic dose at baseline mean ± SD CPZE/mg per day	219.41 ± 184.85
Antidepressant medication: N (Y / N)	12 / 25
GABAergic medication: N (Y / N)	18/19
GABAergic medication: N (BDZ/Val/Li/Zop/PreG/Lam)	11/7/3/1/1/1
Current tobacco use N (Y / N)	15 / 22
Current alcohol current use N (Y / N)	10 / 27
Substance abuse ever N (Y / N)	25 / 12
Substance abuse ever by type (N):	
Cannabis	23
Cocaine	13
Amphetamines	11
Hallucinogens	8
Ketamine	6
Legal Highs	5
Inhalants	2
Heroin	1
Current Substance use N, (Y / N)	7 / 30
Current Substance use type (N)	
Cannabis	6
Cocaine	4
Amphetamines	1
Hallucinogens	0
Ketamine	0
Legal Highs	0
Inhalants	0
Heroin	0

*Note*: Values are provided as mean ± SD unless otherwise stated. CGI-S, Clinical Global Impression- Schizophrenia; CPZE, Chlorpromazine equivalent dose; GAF, Global Assessment of Functioning; PANSS, Positive and Negative Syndrome Scale; PSP, Personal and Social Performance. Diagnosis, F20, Schizophrenia; F25, Schizoaffective disorder; CNS medication, 5-HT, serotonergic antidepressants (including citalopram, duloxetine, escitalopram, fluoxetine, mirtazapine, sertraline, reboxetine, venlafaxine); GABA, GABAergic medication (including BDZ, benzodiazepines; Val, valproate; Li, lithium; Zop, zopiclone; PreG, pregabalin; Lam, lamotrigine).

Symptom severity and functioning were improved after 12 weeks of clozapine compared to baseline (table 2). At 12 weeks, the mean plasma clozapine level was 0.46 ± 0.28 ng/ml (supplementary table 1). The percentage change in PANSS total score was associated with clozapine dose at 12 weeks (*r* = −.50, *n* = 30, *P* = .01), likely reflecting the prescription of higher doses to individuals showing less symptomatic improvement. The change in total PANSS score was not related to plasma clozapine level at 12 weeks (*P* > .05).

**Table 2.** Symptoms and Functioning at Baseline and 12 Weeks

Symptoms and Functioning			
	Baseline	12 Weeks	
Total sample (Baseline *N* = 37; 12 Weeks *N* = 30)			
PANSS—Positive	18.00 ± 5.87	13.57 ± 4.85	
PANSS—Negative	19.22 ± 7.52	15.37 ± 6.20	
PANSS—General	35.04 ± 7.23	26.80 ± 6.08	
PANSS—Total	72.19 ± 15.01	55.77 ± 14.26	
CGI-S (median; range; min; max)	5; 3; 4; 7	4; 3; 3; 6	
GAF	46.22 ± 10.45	59.90 ± 10.35	
PSP	52.27 ± 14.80	62.27 ± 10.85	
Sample with PANSS at both timepoints (*N* = 30)			
PANSS—Positive	18.00 ± 6.17	13.57 ± 4.85	*T*(29) = 6.68; *P* < .01
PANSS—Negative	18.30 ± 7.17	15.37 ± 6.19	*T*(29) = 3.47; *P* < .01
PANSS—General	34.03 ± 7.09	26.80 ± 6.08	*T*(29) = 7.06; *P* < .01
PANSS—Total	70.33 ± 15.51	55.77 ± 14.26	*T*(29) = 7.71; *P* < .01
CGI-S (median; range; min; max)	5; 3; 4; 7	4; 3; 3; 6	*Z* = 4.36; *P* < .01
GAF	47.13 ± 10.72	59.90 ± 10.35	*T*(29) = 5.92; *P* < .01
PSP	52.97 ± 15.33	62.27 ± 10.85	*T*(29) = 3.64; *P* < .01

*Note*: Values are provided as mean ± SD unless otherwise stated. CGI-S, Clinical Global Impression- Schizophrenia; GAF, Global Assessment of Functioning; PANSS, Positive and Negative Syndrome Scale; PSP, Personal and Social Performance Scale.

### ^1^H-MRS Spectral Quality

Of the 35 baseline ^1^H-MRS sessions, all ACC spectrum were included in the analyses, but Glu and Glx values were excluded in one caudate spectra due to CRLB > 20%. Of the 27 12-week ^1^H-MRS sessions, CRLB > 20% led to exclusion of Glu and Glx in one ACC spectra and Glx but not Glu in one caudate spectra. At 12 weeks, ACC but not caudate spectrum were acquired in 2 participants. This resulted in ACC glutamate values for 35 patients at baseline, 26 at 12 weeks, and 24 at both timepoints, and caudate glutamate values for 34 patients at baseline, 25 at 12 weeks, and 23 at both timepoints. All values for metabolites, spectral quality, and voxel tissue composition are presented in supplementary tables 2 and 3. GM ratio of the ACC voxel was significantly lower at 12 weeks compared to at baseline. There were no significant differences between timepoints in caudate voxel tissue composition (supplementary table 2).

### Baseline Glutamate Metabolite Levels and Sample Characteristics

Baseline glutamate metabolite levels in the ACC and caudate were not associated with age, duration of illness, age at illness onset, sex, current smoking status, presence of GABAergic medication or voxel GM ratio (*P* > .05). Baseline ACC Glu_corr_ was higher in clozapine naïve participants compared to those who had previously taken clozapine (mean ± SD: ACC Glu_corr_ naïve: 14.80 ± 2.11, *n* = 29; retrial: 12.62 ± 3.12, *n* = 6; *t*(33) = 2.12; *P* = .04). ACC Glx_corr_ showed a similar effect of previous clozapine exposure (naïve: 20.73 ± 3.82, *n* = 29; retrial: 16.13 ± 4.48, *n* = 6; *t*(33) = 2.50; *P* = .02). The elevation in ACC glutamate metabolites in clozapine naïve compared to retrial participants remained significant when covarying for ACC GM ratio (Glu_corr_: *F*(32) = 4.29; *P* = .05; Glx_corr_: *F*(32) = 5.85; *P* = .02). Caudate Glu_corr_ did not differ between clozapine naïve and retrial participants (*P* > .6). Baseline ACC Glu_corr_ was also higher in participants who reported drinking alcohol compared to those who did not (ACC Glu_corr_ non-alcohol drinkers 13.82 ± 2.18, *n* = 26; alcohol drinkers: 16.19 ± 2.26, *n* = 9; *t*(33) = 2.78; *P* = .01), including when covarying for ACC GM ratio (*F*(32) = 8.44; *P* = .01). This effect was present at trend-level for ACC Glx_corr_ (*P =* .08). Caudate Glu_corr_, was higher in participants prescribed antidepressants at baseline (Caudate Glu_corr_ no antidepressant 11.18 ± 1.98, *n* = 24; antidepressant: 14.23 ± 5.72, *n* = 10; *t*(32) = 2.34; *P* = .03), for caudate Glx_corr_ this effect had a significance value of *P* = .13.

### Effect of 12-Week Clozapine on Glutamate Levels

**Fig. 1.**
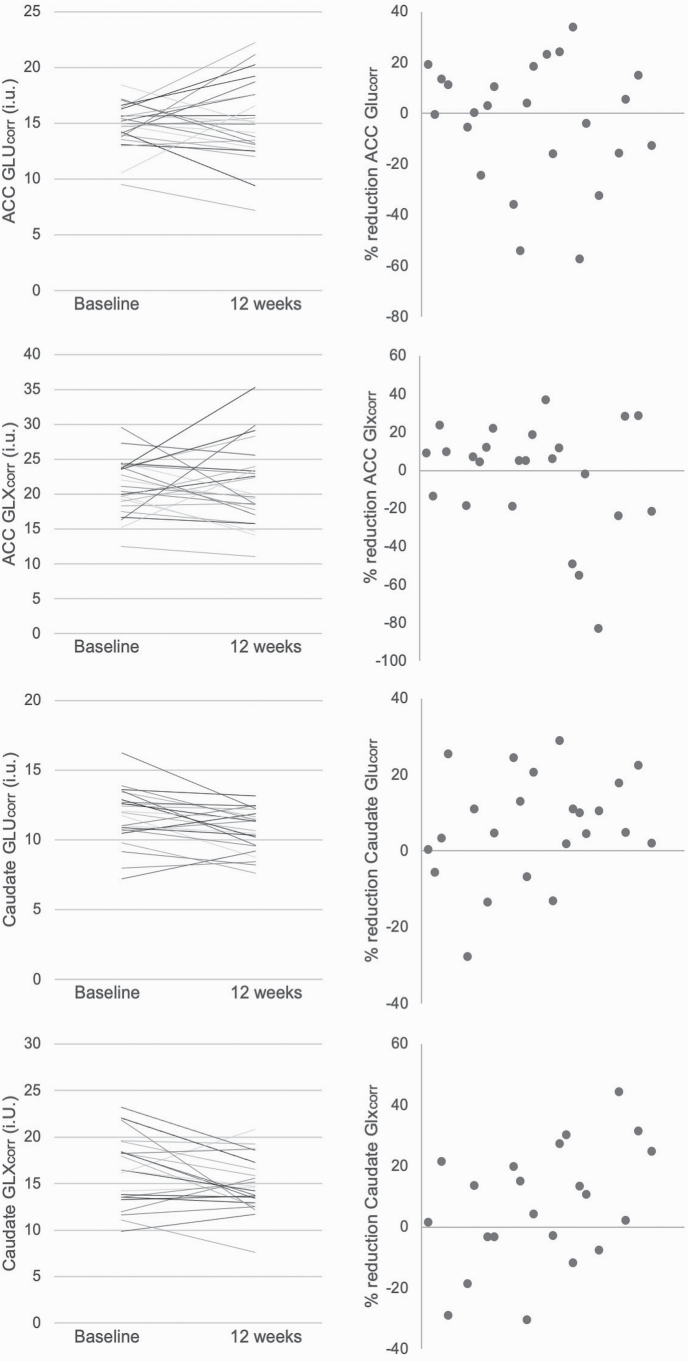
Values in individual participants for Glutamate (Glu_corr_) and Glx (Glx_corr_, glutamate plus glutamine) in the anterior cingulate cortex (ACC) and right caudate before and 12 weeks after switching to clozapine (left). The right column presents data as percentage reduction. In the caudate, Glu_corr_ was significantly reduced after 12 weeks of clozapine treatment (*P* = .01).

In the ACC, there was no significant difference in Glu_corr_ or Glx_corr_ at baseline compared to after 12 weeks of clozapine treatment (table 3, figure 1). This was also the case when analysis was restricted to participants who were clozapine naïve at baseline, or who did not drink alcohol, or when covarying for the numerical difference in voxel GM ratio over time, clozapine dose, or plasma levels at 12 weeks (*P* > .05).

**Table 3.** Glutamatergic Metabolites in the Anterior Cingulate Cortex and Right Caudate at Baseline and After 12 Weeks of Clozapine in Participants With Treatment-Resistant Schizophrenia

	Anterior Cingulate Cortex		
Metabolite	Baseline	12 Weeks	Repeated Measures ANOVA
Total sample			
Glu_corr_	14.43 ± 2.41 (35)	15.46 ± 3.68 (26)	
Glx_corr_	19.94 ± 4.42 (35)	21.40 ± 5.57 (26)	
Completed sample			
Glu_corr_	14.77 ± 1.99 (24)	15.15 ± 3.58 (24)	*F* (1,23) = 0.02, *P* = .59
Glx_corr_	21.05 ± 3.95 (24)	21.15 ± 5.68 (24)	*F* (1,23) = 0.01, *P* = .93
	Right Caudate Nucleus		
Metabolite	Baseline	12 Weeks	
Total sample			
Glu_corr_	12.08 ± 3.69 (34)	10.85 ± 1.58 (25)	
Glx_corr_	17.39 ± 5.56 (34)	14.90 ± 2.81 (25)	
Completed sample			
Glu_corr_	11.66 ± 2.00 (23)	10.73 ± 1.56 (23)	***F* (1,22) = 7.61, *P* = .01**
Glx_corr_	16.34 ± 3.75 (22)	14.78 ± 2.96 (22)	*F* (1,21) = 4.73, *P* = .04

*Note*: Data is provided for the total number of subjects in which ^1^H-MRS was available each timepoint (Total sample) and for the sample who completed ^1^H-MRS at both timepoints (Completed Sample). Data are presented as mean ± SD (*n*). Glu, Glutamate; Glx, Glutamate + Glutamine. The reduction in caudate Glucorr was significant after multiple comparisons correction (bold).

In the caudate, Glu_corr_ was significantly reduced after 12 weeks of clozapine treatment compared to at baseline (table 3, figure 1). The reduction in caudate Glu_corr_ remained significant when covarying for numerical difference in caudate voxel GM ratio over time (*P* = .005), antidepressant use at baseline (*P* = .02), clozapine dose (*P* = .05), or clozapine plasma levels at 12 weeks (*P* = .003). Caudate Glx_corr_ showed a similar pattern of reduction over time, but this was not significant following multiple comparisons correction (table 3).

### Relationships Between Glutamatergic Metabolites and Symptoms

**Fig. 2.**
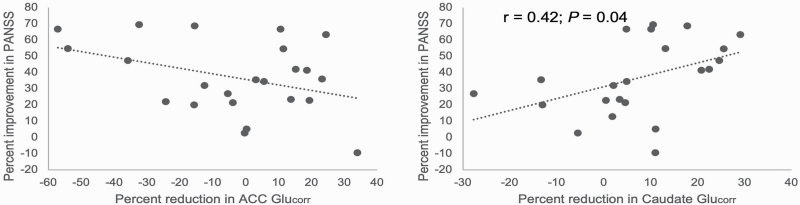
Relationships between the percentage reduction in Glu_corr_ in the anterior cingulate cortex (ACC) and caudate and the percentage improvement in PANSS total scores 12 weeks after switching to clozapine treatment. The percentage reduction in symptoms was not significantly correlated with the percentage reduction in Glu_corr_ in the (ACC), (*n* = 24; *r* = −.36; *P* = .08). In the caudate, greater reductions in Glu_corr_ were associated with greater improvements in symptoms (*n* = 23, *r* = .42, *P* = .04).

The relationships between percentage change in ACC glutamate metabolites and percentage change in PANSS total score were nonsignificant (ACC Glu_corr_: *n* = 24; *r* = −.36; *P* = .08; figure 2). In the caudate, there was a direct relationship, such that greater reductions in Glu_corr_, but not Glx_corr_ were associated with greater improvements in symptoms (*n* = 23, *r* = .42, *P* = .04), which was also significant when controlling for numerical difference in caudate GM ratio over time (*P* = .03). Relationships between baseline Glu_corr_ and Glx_corr_ and total PANSS score at baseline or 12 weeks were nonsignificant.

## Discussion

Although modulation of brain glutamate has been suggested to contribute to the efficacy of clozapine, this has only been supported by indirect evidence.^30,41–43^ In this study, we examined glutamate levels in the ACC and caudate in patients with TRS before and after 12 weeks of switching to clozapine treatment. We found that reductions in glutamate levels in the caudate over this period, and that this reduction was associated with improvements in symptoms. In contrast, there was no change in glutamatergic metabolites in the ACC. Secondary findings included observations that ACC glutamate was higher in clozapine naïve than re-trial participants, and in those who had consumed alcohol in the last week, and that caudate glutamate at baseline was higher in patients who were also taking an antidepressant medication. Overall, our main findings indicate that glutamate reductions in the caudate, but not ACC, occur over the first months of clozapine treatment.

We hypothesized that clozapine would reduce ACC glutamate levels, on the basis of preclinical research,^19–21^ and because the elevations ACC glutamate levels which have been observed in patients who have responding poorly to antipsychotics^4,5,7,8^ are not apparent in patients who are taking and have responded to clozapine.^9,10^ Contrary to this hypothesis, however, we did not observe reductions in ACC glutamate over 12 weeks of clozapine treatment, suggesting ACC glutamate levels are relatively stable over the initial months following clozapine initiation. It should be noted that previous cross-sectional studies of patients who were taking clozapine included participants who were already stabilized on clozapine therapy for several years.^9,10^ Although the number of patients in our study who had taken clozapine previously was small, this subsample had lower ACC glutamate at baseline compared to those who were clozapine naïve. This could indicate that reductions in ACC glutamate metabolites might be observed after longer-term clozapine exposure. A second interpretation is that reductions in ACC glutamate are only observed in patients who have minimal symptom severity after clozapine treatment. In the study of Iwata,^10^ ACC Glx did not differ between healthy volunteers and clozapine-treated patients with CGI-S and positive symptom scores of mild or less. While on average symptoms improved during our study, 83% (25/30) of participants still had CGI-S scores of ≥4 (moderate severity) at 12 weeks, so were relatively more unwell.

In the caudate, glutamate reduced over 12 weeks of clozapine treatment, and this reduction correlated with the degree of symptomatic improvement. Reductions in glutamate metabolites in the caudate are also observed over 4 weeks of antipsychotic treatment in first-episode psychosis.^13^ The caudate nucleus modulates information flow through corticostriatal loops to control cognitive and emotional behavior.^44^ It is one of the major regions implicated in schizophrenia and in the action of dopamine D2 receptor antagonist antipsychotics.^45,46^ Clozapine could decrease caudate glutamate levels via its interactions with multiple receptor subtypes in cortical as well as subcortical regions^47^ as well as potentially through more direct effects on the glutamatergic system.^24,48–51^ Given evidence for cortical abnormalities in TRS,^52^ studies examining corticostriatal connectivity during clozapine treatment would be useful to investigate these mechanisms further. As the ^1^H-MRS signal includes all MR-visible glutamate in the voxel, future pharmacological studies in animals combining ^1^H-MRS with invasive techniques to examine neurotransmission may provide further mechanistic information.

A secondary and exploratory finding was that ACC glutamate was elevated in patients who reported consuming alcohol in the week prior to imaging, compared to those who did not. We are not aware of previous research examining the effects of alcohol intake on brain glutamate levels in patients with schizophrenia specifically. ^1^H-MRS studies in alcohol use disorder have reported increases in ACC glutamate during initial (24 hour) withdrawal but reductions during drinking or extended abstinence.^53–55^ We excluded participants meeting the criteria for current substance dependence, as is common within schizophrenia neuroimaging studies. Our findings indicate that a more detailed analysis of the potential effects of alcohol intake may be warranted, including intake below threshold levels for dependence. Similarly, the observation that caudate glutamate levels were higher in patients who were taking an antidepressant highlights the importance of considering potential influences of concomitant medications. Nonetheless, our main findings of no change in ACC glutamate and a reduction in caudate glutamate over clozapine treatment did not change when alcohol or antidepressant use were included as variables in the respective analyses.

Strengths of our study include the longitudinal design and our ability to recruit a relatively unwell patient group at the time of clozapine initiation. Our study had provision to include patients who lacked mental capacity to consent, in order to aid the generalizability of the research to the clinical population. However, a significant proportion of the total sample were unable or declined to participate in MRI. This may impact on the generalizability of the MRI study, including potentially biasing results towards less unwell TRS patients who may also have been more likely to respond to clozapine. As participants had significant illness and clozapine is the only recommended antipsychotic for TRS, we did not investigate the effects of clozapine on glutamate in comparison to placebo or an alternative active compound. These comparisons would be required to specifically attribute the reductions in symptoms or caudate glutamate levels to clozapine treatment rather than nonspecific aspects of study participation. A general limitation of ^1^H-MRS at 3 Tesla is that the glutamate signal has an estimated contamination by glutamine, albeit of <10%, and that glutamine alone cannot be reliably estimated.^4,56^ Findings should also be interpreted in the context that ^1^H-MRS measures the total amount of intracellular glutamate, and the voxels include contributions from gray matter, white matter, and CSF. High bandwidth Very Spatially Selective Outer Volume Suppression pulses were used to suppress signal from outside the voxel, which may have led to underestimation of the unsuppressed water peak and overestimation of metabolite concentrations.^57^

In summary, the main finding of this study is that ^1^H-MRS measures of glutamate metabolites in the caudate were reduced over 12 weeks of clozapine treatment and that this was associated with symptom reduction in TRS. In contrast, ACC glutamate was not significantly altered by clozapine over the observation period. Future studies could examine whether reductions in ACC glutamate occur over a longer period of clozapine treatment and the relationship with clinical outcome and could also examine corticostriatal connectivity to provide deeper mechanistic insight.

## Supplementary Material

sbaa156_suppl_Supplementary-Material
