# Supplementary material for: Changes in Brain Glutamate on Switching to Clozapine in Treatment-Resistant Schizophrenia
Source: Schizophr Bull. 2021 Jan 5;47(3):662–71. doi: 10.1093/schbul/sbaa156 (PMC8084451; doi:10.1093/schbul/sbaa156)
Supplement: sbaa156_suppl_Supplementary-Material [file sbaa156_suppl_supplementary-material.pdf]

Supplemental Information

McQueen et al., Changes in Brain Glutamate on Switching to Clozapine in Treatment Resistant Schizophrenia.

Supplementary Methods

MR data were acquired on a 3 Tesla MR750 scanner (General Electric, Chicago, USA) using a 12 channel head, neck and spine coil. The scanning session commenced with a localizer, standard axial T2-weighted fast spin echo and FLAIR scan, with a 3D T1-weighted structural scan (TR / TE = 7.312 / 3.01). ^{1}H-MRS spectra were acquired in 8 cm^3 (2 x 2 x 2 cm^3) voxels prescribed in the bilateral ACC[1, 2] and in the right caudate nucleus.[3, 4] The caudate voxel in the right head of striatum with the lower end of the voxel 3mm dorsal to the anterior commissure, to include the maximum amount of grey matter and minimum amount of CSF (Figure S1). Images of voxel placement at baseline were used to assist with within-subject consistency of voxel placement at follow-up. Spectra were acquired using a conventional PRESS (Point RESolved Spectroscopy) acquisition with 96 averages, TR = 3000 ms and with a TE = 30 ms in the ACC and a TE = 35 ms in the caudate,[4] which a pilot scan identified as optimal for each voxel on our MR system. We used the standard GE PROBE (PROton Brain Exam) sequence which uses a standardized chemically selective suppression (CHESS) water suppression routine. Unsuppressed water spectra (16 averages) were used for eddy current correction and water scaling.

Data processing

Spectra were analyzed with LCModel version 6.3-0I using the standard basis set of 16 metabolites (L-alanine, aspartate, creatine, phosphocreatine, GABA, glucose, glutamine, glutamate, glycerophosphocholine, glycine, myo-inositol, L-lactate, N-acetylaspartate, N-acetylaspartylglutamate, phosphocholine, taurine) experimentally acquired at the same field strength and TE as the corresponding voxel and provided by the software's author.[5] The spectral window for fitting was 0.2-4.0 ppm. Spectral quality was evaluated by visual inspection, and individual metabolite estimates were excluded if the Cramér Rao Lower Bound estimates of the standard deviations (%SD) were greater than >20%.

To calculate ^{1}H-MRS voxel tissue content, the T1-weighted structural images were segmented into grey matter, white matter and cerebrospinal fluid (CSF) using Statistical Parametric Mapping 8 (SPM-8) (http//www.fil.ion.ucl.ac.uk/spm) running in Matlab 6.5 (Mathworks Inc. Sherbon MA, USA). GE Sage 7 software (GE Medical Systems) was used to locate the coordinates of each voxel, which were then mapped on to the segmented structural image using in-house software. Metabolite values were corrected for voxel tissue content using the formula:

Mcorr = M*(WM + 1.21*GM + 1.55* CSF) / (wm + gm),

where M is the uncorrected metabolite, and WM, GM and CSF indicate the fraction of white and grey matter and cerebrospinal fluid content in the voxel. The formula assumes a CSF water concentration of 55,556mol/m3.[6, 7] Voxel GM ratio was calculated as GM/(GM+WM).

	**6 weeks (N = 30)**	**12 weeks (N = 28)**
Plasma clozapine (ng/mL)	0.33 ± 0.20	0.46 ± 0.28
Plasma norclozapine (ng/mL)	0.18 ± 0.10	0.23 ± 0.11
Clozapine dose (daily mg)	288.75 ± 134.13	324.17 ± 144.66

Supplement Table 1. Clozapine dose and plasma clozapine and norclozapine levels at 6- and 12-weeks following clozapine initiation. Data are provided as mean ± standard deviation. Eighteen participants had plasma clozapine levels above 350ng/mL for at least 4 weeks prior to the 12-week scan, 11 had below threshold levels for this period and 8 participants had missing data.

Anterior Cingulate Cortex	Baseline<br>T: n = 35<br>C: n = 24	Follow-up<br>T: n = 26<br>C: n = 24	Significance<br>(P value)
Line-width, FWHM (T)	0.04 ± 0.01	0.06 ± 0.05	
Line-width, FWHM (C)	0.04 ± 0.01	0.06 ± 0.07	0.19
Signal to noise ratio (T)	23.17 ± 3.75	21.33 ± 4.84	
Signal to noise ratio (C)	22.72 ± 3.43	21.2 ± 4.93	0.12
Voxel WM (T)	0.13 ± 0.04	0.13 ± 0.05	
Voxel WM (C)	0.11 ± 0.35	0.13 ± 0.05	0.09
Voxel GM (T)	0.58 ± 0.09	0.53 ± 0.07	
Voxel GM (C)	0.59 ± 0.09	0.54 ± 0.07	<0.01
Voxel CSF (T)	0.29 ± 0.11	0.34 ± 0.08	
Voxel CSF (C)	0.30 ± 0.10	0.33 ± 0.08	0.05
Voxel GM ratio (T)	0.81 ± 0.06	0.81 ± 0.06	
Voxel GM ratio (C)	0.84 ± 0.04	0.81 ± 0.06	0.01
Glu %SD (T)	7.03 ±1.60	7.35 ± 2.76	
Glu %SD (C)	6.83 ± 1.43	7.42 ± 2.86	0.25
Glx %SD (T)	7.37 ± 1.65	7.69 ± 1.72	
Glx %SD (C)	6.96 ± 1.40	7.75 ± 1.78	0.05
NAA %SD (T)	3.17 ± 0.62	3.22 ± 0.89	
NAA %SD (C)	3.24 ± 0.66	3.28 ± 0.89	0.86
TCho %SD (T)	3.03 ± 0.50	3.22 ± 0.63	
TCho %SD (C)	3.04 ± 0.45	3.24 ± 0.66	0.13
mI %SD (T)	5.46 ± 2.66	5.67 ± 2.66	
mI %SD (C)	5.68 ± 3.06	5.72 ± 2.76	0.89
Cr %SD (T)	2.77 ± 0.43	2.93 ± 0.62	
Cr %SD (C)	2.84 ± 0.37	2.92 ± 0.64	0.60
**Caudate Nucleus**	**Baseline**<br>**T: n = 34**<br>**C: n = 23**	**Follow-up**<br>**T: n = 25**<br>**C: n = 23**	
Line-width, FWHM (T)	0.07 ± 0.01	0.08 ± 0.02	
Line-width, FWHM (C)	0.07 ± 0.01	0.08 ± 0.02	0.19
Signal to noise ratio (T)	16.74 ± 4.28	17.36 ± 4.23	

Signal to noise ratio (C)	17.78 ± 3.95	17.13 ± 4.31	0.42
Voxel WM (T)	0.46 ± 0.09	0.47 ± 0.09	
Voxel WM (C)	0.47 ± 0.07	0.46 ± 0.09	0.66
Voxel GM (T)	0.49 ± 0.08	0.50 ± 0.07	
Voxel GM (C)	0.50 ± 0.09	0.51 ± 0.09	0.73
Voxel CSF (T)	0.05 ± 0.06	0.02 ± 0.03	
Voxel CSF (C)	0.03 ± 0.03	0.03 ± 0.03	0.67
Voxel GM ratio (T)	1.46 ± 0.09	1.47 ± 0.09	
Voxel GM ratio (C)	1.47 ± 0.07	1.46 ± 0.09	0.80
Glu %SD (T)	9.17 ± 5.10	8.16 ± 1.76	
Glu %SD (C)	8.13 ± 2.49	8.26 ± 1.81	0.78
Glx %SD (T)	10.64 ± 5.52	10.20 ± 2.33	
Glx %SD (C)	10.26 ± 3.21	10.35 ± 2.37	0.91
NAA %SD (T)	3.75 ± 1.39	3.44 ± 1.11	
NAA %SD (C)	3.61 ± 1.34	3.48 ± 1.16	0.67
TCho %SD (T)	3.94 ± 1.48	3.67 ± 1.07	
TCho %SD (C)	3.65 ± 0.98	3.72 ± 1.11	0.65
mI %SD (T)	9.42 ± 3.40	8.24 ± 2.39	
mI %SD (C)	9.45 ± 3.16	8.41 ± 2.50	0.13
Cr %SD (T)	3.49 ± 1.00	3.20 ± 0.82	
Cr %SD (C)	3.30 ± 0.70	3.22 ± 0.85	0.49

Supplement Table 2: Indices of spectral quality in the anterior cingulate cortex and right caudate nucleus. All data are presented as mean ± standard deviation, for both the total number of subjects in which ^{1}H-MRS was available each time-point (T), and in the sample who completed ^{1}H-MRS at both timepoints (C). The P values relate to the significance of paired samples analysis in participants who completed 1H-MRS at both timepoints. Abbreviations: Cr: Creatine; %SD: Cramer Rao Lower Bound estimate of the standard deviation; CSF: Cerebral spinal fluid; FWHM: full width at half maximum, as reported by LCModel output; Glu: Glutamate; Glx: Glutamate plus Glutamine; GM: Grey matter; GM ratio: (GM/GM+WM); NAA: N-acetyl aspartate plus N-acetylaspartyl-glutamate; TCho, Total Choline (choline plus phosphocholine); mI: myo-inositol; WM: white matter.

**Anterior Cingulate Cortex**	**Baseline**<br>**T: n = 35**<br>**C: n = 24**	**Follow-up**<br>**T: n = 26**<br>**C: n = 24**	**Significance**<br>**(P value)**
Glu_{corr} (T)	14.43 ± 2.41	15.46 ± 3.68	
Glu_{corr} (C)	14.77 ± 1.99	15.15 ± 3.58	0.59
Glx_{corr} (T)	19.94 ± 4.42	21.40 ± 5.57	
Glx_{corr} (C)	21.05 ± 3.95	21.15 ± 5.68	0.93
NAA_{corr} (T)	15.40 ± 3.00	16.54 ± 3.10	
NAA_{corr} (C)	15.40 ± 2.68	16.25 ± 2.95	0.16
$TCho_{corr}$ (T)	3.37 ± 0.85	3.53 ± 1.02	
$TCho_{corr}$ (C)	3.41 ± 0.74	3.46 ± 0.99	0.78
mI_{corr} (T)	9.03 ± 2.65	9.58 ± 2.53	
mI_{corr} (C)	8.84 ± 2.15	9.49 ± 2.61	0.16
Cr_{corr} (T)	12.55 ± 2.65	12.85 ± 2.68	
Cr_{corr} (C)	12.41 ± 2.46	12.72 ± 2.65	0.53
Glu (T)	7.76 ± 1.33	7.84 ± 1.71	
Glu (C)	7.91 ± 1.25	7.81 ± 1.77	0.70
Glx (T)	10.77 ± 2.22	10.36 ± 2.90	
Glx (C)	11.07 ± 2.42	10.36 ± 3.01	0.20
NAA (T)	8.24 ± 1.06	8.28 ± 1.22	
NAA (C)	8.23 ± 1.20	8.26 ± 1.26	0.87
TCho (T)	1.80 ± 0.31	1.75 ± 0.36	
TCho (C)	1.81 ± 0.28	1.74 ± 0.36	0.27
mI (T)	4.79 ± 1.05	4.79 ± 1.14	
mI (C)	4.70 ± 1.07	4.80 ± 1.17	0.61
Cr (T)	6.66 ± 0.78	6.39 ± 0.94	
Cr (C)	6.55 ± 0.74	6.41 ± 0.96	0.45
**Caudate Nucleus**	**Baseline**<br>**T: n = 34**<br>**C: n = 23**	**Follow-up**<br>**T: n = 25**<br>**C: n = 23**	
Glu_{corr} (T)	12.08 ± 3.69	10.85 ± 1.58	
Glu_{corr} (C)	11.66 ± 2.00	10.73 ± 1.56	**0.01**
Glx_{corr} (T)	17.39 ± 5.56	14.90 ± 2.81	

Glx_{corr} (C)	16.34 ± 3.75	14.78 ± 2.97	**0.04**
NAA_{corr} (T)	11.39 ± 2.99	11.15 ± 1.50	
NAA_{corr} (C)	11.84 ± 1.03	11.11 ± 1.57	**0.02**
$TCho_{corr}$ (T)	2.56 ± 0.72	2.40 ± 0.32	
$TCho_{corr}$ (C)	2.44 ± 0.35	2.40 ± 0.33	0.64
mI_{corr} (T)	4.66 ± 2.18	4.87 ± 1.16	
mI_{corr} (C)	4.28 ± 1.18	4.86 ± 1.19	0.06
Cr_{corr} (T)	9.44 ± 2.63	8.75 ± 1.28	
Cr_{corr} (C)	9.23 ± 1.51	8.76 ± 1.23	0.08
Glu (T)	9.83 ± 1.88	9.68 ± 1.37	
Glu (C)	10.08 ± 1.56	9.58 ± 1.37	0.07
Glx (T)	14.13 ± 3.19	13.31 ± 2.57	
Glx (C)	14.11 ± 2.92	13.18 ± 2.71	0.13
NAA (T)	9.36 ± 1.95	9.95 ± 1.30	
NAA (C)	10.22 ± 0.79	9.91 ± 1.34	0.19
TCho (T)	2.08 ± 0.40	2.05 ± 0.51	
TCho (C)	2.12 ± 0.29	2.05 ± 0.52	0.56
mI (T)	3.76 ± 1.21	4.34 ± 1.02	
mI (C)	3.79 ± 0.88	4.31 ± 1.07	0.03
Cr (T)	7.67 ± 1.41	7.83 ± 1.08	
Cr (C)	7.97 ± 1.18	7.83 ± 1.06	0.49

Supplement Table 3. Metabolite values in the anterior cingulate cortex and right caudate nucleus. The table shows values corrected for voxel tissue fractions (corr) and uncorrected data, for both the total number of subjects in which ^{1}H-MRS was available each time-point (T), and in the sample who completed ^{1}H-MRS at both timepoints (C). All data are presented as mean ± standard deviation. The P values relate to the significance of the repeated measures analysis in participants who completed 1H-MRS at both baseline and 12 weeks (no covariates). **Abbreviations**: Cr: Creatine; Glu: Glutamate; Glx: Glutamate plus Glutamine; NAA: N-acetyl aspartate plus N-acetylaspartyl-glutamate; TCho, Total Choline (choline plus phosphocholine); mI: myo-inositol.

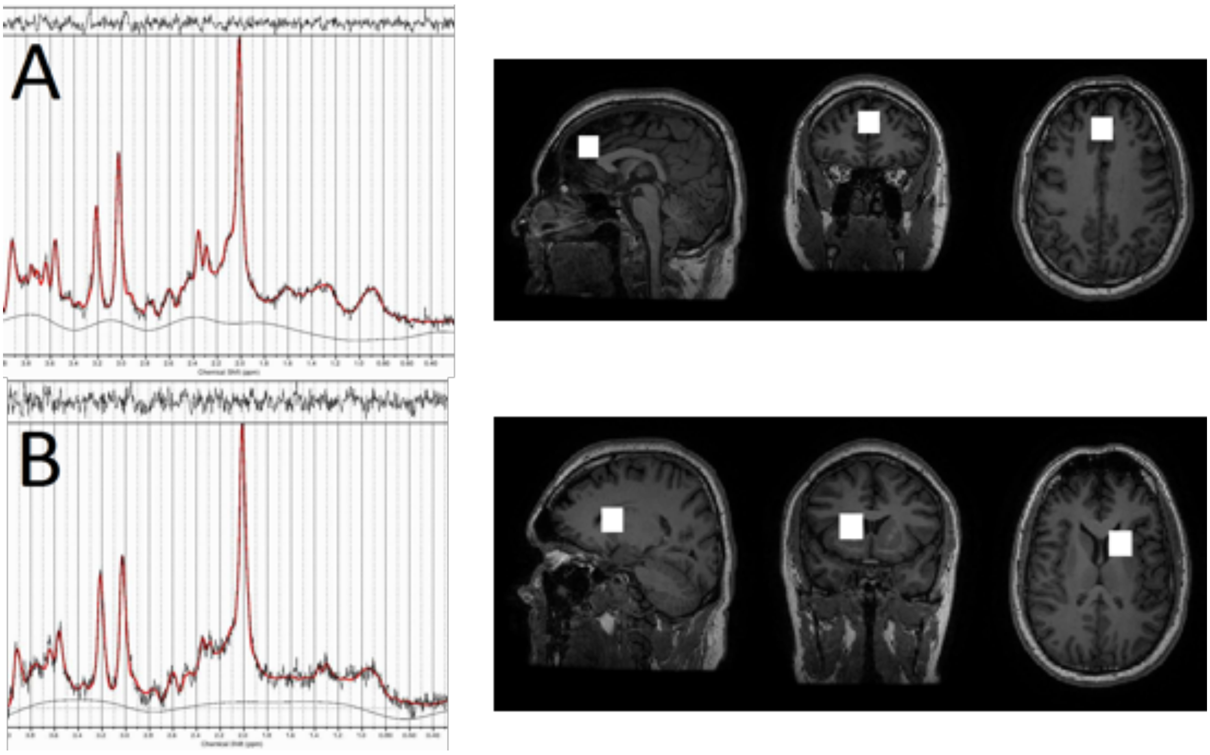

Supplement Figure 1. ^{1}H-MRS example spectra and voxel positioning in the anterior cingulate cortex (ACC, A) and right caudate nucleus (B). The left-hand panels show the LCModel output of the fit (red), overlaid on the acquired spectrum (black). The estimated baseline is shown underneath in black. The right-hand panel shows example voxel placement overlaid on a T1-weighted structural image.

References

1. Egerton A, Brugger S, Raffin M, Barker GJ, Lythgoe DJ, McGuire PK, Stone JM. Anterior Cingulate Glutamate Levels Related to Clinical Status Following Treatment in First-Episode Schizophrenia. *Neuropsychopharmacology* 2012;37(11):2515-2521.

2. Egerton A, Broberg BV, Van Haren N, et al. Response to initial antipsychotic treatment in first episode psychosis is related to anterior cingulate glutamate levels: a multicentre (1)H-MRS study (OPTiMiSE). *Mol Psychiatry* 2018;23(11):2145-2155.

3. McQueen G, Lally J, Collier T, et al. Effects of N-acetylcysteine on brain glutamate levels and resting perfusion in schizophrenia. *Psychopharmacology (Berl)* 2018;235(10):3045-3054.

4. de la Fuente-Sandoval C, Leon-Ortiz P, Azcarraga M, et al. Glutamate levels in the associative striatum before and after 4 weeks of antipsychotic treatment in first-episode psychosis: a longitudinal proton magnetic resonance spectroscopy study. *JAMA psychiatry* 2013;70(10):1057-1066.

5. Provencher SW. Estimation of metabolite concentrations from localized in vivo proton NMR spectra. *Magn ResonMed* 1993;30(6):672-679.

6. Kreis R, Ernst T, Ross BD. Development of the human brain: in vivo quantification of metabolite and water content with proton magnetic resonance spectroscopy. *Magn Reson Med* 1993;30(4):424-437

7. Gasparovic C, Song T, Devier D, et al. Use of tissue water as a concentration reference for proton spectroscopic imaging. *Magn Reson Med* Jun 2006;55(6):1219-1226.
